# Definition of an Indoor Air Sampling Strategy for SARS-CoV-2 Detection and Risk Management: Case Study in Kindergartens

**DOI:** 10.3390/ijerph19127406

**Published:** 2022-06-16

**Authors:** Laura Borgese, Giuseppe Tomasoni, Filippo Marciano, Annalisa Zacco, Fabjola Bilo, Elena Stefana, Paola Cocca, Diana Rossi, Paola Cirelli, Angelo Luigi Camillo Ciribini, Sara Comai, Silvia Mastrolembo Ventura, Michela Savoldi Boles, Diletta Micheletti, Daniela Cattivelli, Serena Galletti, Sophie Dubacq, Maria Grazia Perrone, Laura Eleonora Depero

**Affiliations:** 1 INSTM and Chemistry for Technologies Laboratory, Department of Mechanical and Industrial Engineering, University of Brescia, Via Branze 38, 25123 Brescia, Italy; annalisa.zacco@unibs.it (A.Z.); fabjola.bilo@unibs.it (F.B.); laura.depero@unibs.it (L.E.D.); 2 Smart Solutions S.r.l., Via Corfù, 106, 25124 Brescia, Italy; 3 Department of Mechanical and Industrial Engineering, University of Brescia, Via Branze 38, 25123 Brescia, Italy; giuseppe.tomasoni@unibs.it (G.T.); elena.stefana@unibs.it (E.S.); paola.cocca@unibs.it (P.C.); diana.rossi@unibs.it (D.R.); 4 Department of Information Engineering, University of Brescia, Via Branze 38, 25123 Brescia, Italy; p.cirelli001@unibs.it; 5 Department of Civil and Environmental Engineering, Architecture and Mathematics, University of Brescia, Via Branze 43, 25123 Brescia, Italy; angelo.ciribini@unibs.it (A.L.C.C.); sara.comai@unibs.it (S.C.); silvia.mastrolemboventura@unibs.it (S.M.V.); 6 BIOSIDE S.r.l., Via A. Einstein, 26900 Lodi, Italy; m.savoldi@bioside.it (M.S.B.); d.micheletti@bioside.it (D.M.); 7 AAT-Advanced Analytical Technologies S.r.l., Via P. Majavacca 12, 29017 Fiorenzuola d’Arda, Italy; daniela.cattivelli@aat-taa.eu (D.C.); serena.galletti@aat-taa.eu (S.G.); 8 Bertin Instruments, Brand of Bertin Technologies S.A.S., 10 Bis Avenue Ampère, 78180 Montigny-le-Bretonneux, France; sophie.dubacq@bertin.fr; 9 TCR Tecora S.r.l., Via delle Primule, 16, 20815 Cogliate, Italy; mariagrazia.perrone@tcrtecora.com; 10 XearPro S.r.l., Via delle Primule, 16, 20815 Cogliate, Italy

**Keywords:** COVID-19, risk assessment and control, hazard identification, aerosol transmission, droplet transmission, bioaerosol sampling, sampling plan, standard guideline, school

## Abstract

In the last two years, the world has been overwhelmed by SARS-CoV-2. One of the most important ways to prevent the spread of the virus is the control of indoor conditions: from surface hygiene to ventilation. Regarding the indoor environments, monitoring the presence of the virus in the indoor air seems to be promising, since there is strong evidence that airborne transmission through infected droplets and aerosols is its dominant transmission route. So far, few studies report the successful detection of SARS-CoV-2 in the air; moreover, the lack of a standard guideline for air monitoring reduces the uniformity of the results and their usefulness in the management of the risk of virus transmission. In this work, starting from a critical analysis of the existing standards and guidelines for indoor air quality, we define a strategy to set-up indoor air sampling plans for the detection of SARS-CoV-2. The strategy is then tested through a case study conducted in two kindergartens in the metropolitan city of Milan, in Italy, involving a total of 290 children and 47 teachers from 19 classrooms. The results proved its completeness, effectiveness, and suitability as a key tool in the airborne SARS-CoV-2 infection risk management process. Future research directions are then identified and discussed.

## 1. Introduction

Many efforts have been made so far to fight the spread of SARS-CoV-2, from tracking infected people and their contacts, imposing restrictions such as lockdowns and social distancing, and adopting protective equipment, to vaccines. The objective of such measures is to interrupt the virus transmission, which have been proved to occur in different ways: close contact through large droplet spray, indirect contact via contaminated objects, and inhalation of infected saliva aerosol particles [1,2,3,4,5,6]. Several approaches to detect the presence of biological agents in indoor environments are available; one of the most used in the case of SARS-CoV-2 is surface sampling. Strong evidence from case and cluster reports indicates that airborne transmission by droplet and aerosol particles is dominant [6], with proximity between people and ventilation of indoor environments being key determinants of transmission risk [7,8]. Therefore, the possibility of directly sampling its presence in the air seems to be significant.

Several studies analyse the ventilation, both natural and mechanical, the use of face masks and their potential effectiveness in controlling the SARS-CoV-2 airborne transmission [3,9], or the use of CO_2_ concentrations in the air as a proxy of infection risk [10]. Nevertheless, few studies attempt to directly detect the presence of the virus in the indoor air [11,12,13,14,15,16]. However, more efforts should be directed towards this weakness in the scientific literature, since any confirmation of the presence of the virus could guide the adoption of more timely, focused, and therefore effective risk control measures. Indeed, the detection of the presence of the virus represents the phase of “risk identification” in the general risk management framework [17] or the phase of “hazard identification” in the context of risk management for health and safety [18].

In addition, some of the other measures put in place have partially failed. This is the case of contact tracing implemented in Italy on a voluntary basis [19], and further underlines the need of effective tools for environmental surveillance based on air sampling.

The literature gap highlighted above is partly due to the fact that the airborne SARS-CoV-2 is not easily detectable, and the setup of an air sampling plan within a real building is also not straightforward.

A sampling plan should take into account a large number of factors regarding the activities carried out in the building, the level of occupancy, the ventilation system, the used air sampling equipment, and other critical parameters [7,20,21]. To set-up such a plan, it is necessary to follow a strategy that defines who, what, where, how, how many, when and how long to sample the air.

In this sense, a standard to guide the definition of an air sampling plan has not yet been developed. Indeed, to the best of our knowledge, the only standard for environmental sampling of SARS-CoV-2 is the protocol published by World Health Organisation (WHO), but it only deals with surface sampling for health care and public health professionals [22]. The lack of standardised air sampling methods and strategies is the case for all biological agents. In fact, for example, European legislation for the assessment and control of risks related to exposure to biological agents at work prescribes “testing” for their presence only “where it is necessary and technically possible”, without any further detail [23,24]. Even the most recent examples of guidelines for indoor air quality control in environments such as schools exclude their applicability to biological agents [25]. On the other hand, the legislation provides more specific guidance on methods and strategies for sampling other airborne contaminants such as chemical agents [23,26].

As a consequence, the primary objective of this paper is to propose a strategy of general applicability that could represent a standard in defining sampling plans to detect the presence of SARS-CoV-2 in the air. Such standard could have a dual purpose: (1) to guide practitioners in designing and carrying out air sampling activities, and (2) to allow for consistent results from different samplings, i.e., samplings carried out in different environments, conditions and geographic locations. This second goal would make such results more suitable for scientific research aimed, for example, at quantifying the airborne virus detection threshold limit for a specific sampler, at identifying reliable proxies, at developing infection risk assessment models, at identifying risk control measures and/or evaluating their effectiveness.

Another objective of this paper is to test the applicability of the proposed sampling strategy in real environments, to verify its completeness and to evaluate its effectiveness in guiding the definition of a sampling plan, through a case study carried out in kindergartens.

The remainder of this paper is organised as follows. In Section 2, we describe the materials and methods adopted for this research, with specific reference to the bioaerosol sampling, the air sampling strategy, and the case study carried out. The results are presented in Section 3, both in terms of the sampling strategy proposed and the findings emerged from the sampling campaign realised within the case study, and discussed in Section 4. Study limitations and future research directions are reported in Section 5, while concluding remarks are provided in the final section.

## 2. Materials and Methods

In order to pursue the objective of proposing a sampling strategy to detect the presence of airborne SARS-CoV-2 in indoor environments, we analysed the state-of-the-art of air sampling in work and living indoor environments, including scientific literature, legislation, technical standards, and guidelines from relevant public agencies and professional associations. In particular, we focused on (a) the techniques and devices for bioaerosol sampling, and (b) the strategies for air sampling in specific contexts or for air sampling of other substances.

### 2.1. Bioaerosol Sampling

With reference to bioaerosol sampling, various techniques and devices have been proposed by occupational health organizations [27]. Several bioaerosol sampling devices are available nowadays and can be grouped in passive and active samplers divided into two categories: dry and wet sampling methods. In passive samplers, particles are collected by gravitational sedimentation. In active samplers, volumes of air are drawn into a sampling inlet and the particle collection can be based on filtering systems (fibre filters, polytetrafluoroethylene filters and gelatine filters), impactors (Andersen impactor, centrifugal impactor), liquid impingers, and cyclone sampler [28,29]. The selection of the device is tricky and its use requires highly qualified personnel [30]. Moreover, a validation in the laboratory by model experiments is necessary to define airborne virus detection threshold limits depending on the aerosol size distribution, sampling efficiency, and sensitivity of the following molecular biology or microbiological tests, e.g., [31,32,33].

Despite the existing methodologies, no method has been recommended for the sampling and determination of viruses in the air, since viruses are not able to grow in general sampling media because of their structure [34]. Depending on the target type of virus, different kinds of sampling devices and parameters should be considered (i.e., sample pump, sampling volume, sampling time) with other factors related to the following detection method (i.e., culture medium and incubation conditions), as it important to maintain the integrity of virus nucleic acid that can rapidly be degraded during the sampling process [35].

Most of the available air sampling devices have not been designed to collect infectious viruses from the air; moreover, their collection efficiency varies depending on the aerosol size distribution and the sampling parameters like the flow rate [33,36]. For this reason, it is important know to characterise the system under investigation at most to maximize the probability of virus collection.

Aerosols produced by human beings have been characterised [20]. It is reported that larger particles are generated by coughing and sneezing [37,38] and smaller particles are emitted during speaking. The latter ones may be also formed by secondary processes such as particle aging or evaporation and may travel up to 1.83 m [39]. Recent studies are focusing to determine the SARS-CoV-2 viral loads within coarse (>5 μm) and fine (≤5 μm) respiratory aerosols to better understand how airborne transmission occurs [40]. Recent literature also reports successful methods that have been used for sampling and detection of SARS like viruses in the air [41].

Filter material plays a key role in collection of viral particle size. Among the filter typologies, polytetrafluoroethylene (PTFE) and gelatine filters [42] showed the highest efficiency [43]. Although filters reveal a high collection efficiency for particles larger than 0.5 µm and are easily handled, the desiccation of bioaerosol particles may occur. This drawback is overcome by other samplers like impingers and cyclones because the collection in liquid prevents particles from drying out. The limitation of the latter sampler is related to the turbulences in the liquid caused by the air that may reduce the viability of particles [36].

Liquid impingers have traditionally been employed for bioaerosol sampling, thanks to the advantage of sample collected in liquid media, which is required in most of the biological analysis [44]. Nevertheless, these samplers demonstrate a reduced collection efficiency and viability of particles due to the evaporation and the re-aerosolisation of collected particles [45,46]. Single-stranded RNA (ssRNA) and double-stranded DNA (dsDNA) were used to determine the collection efficiency of the All Glass Impinger 30, the SKC Bio Sampler^®^ (SKC Inc., Eighty Four, PA, USA), and a frit bubbler for ultrafine particles as a function of particle size, sampler flow rate, and sampling time [47]. In contrast, there is no evidence about the collection efficiency of liquid impingers for ultrafine and sub micrometre particles with diameters < 300 nm. This type of sampler is not recommended for a sampling virus at low viral load because of its limited flowrate capacity. Moreover, foam production during sampling is listed as another restriction of impingers’ samples [48].

Cyclone sampler is another well-studied category of a bio sampler employed for virus air sampling [27,49,50,51], and they have been reported suitable for SARS-CoV-2 collection in hospital rooms during the pandemic time [12].

Devices that use condensation growing tubes as a sample collection strategy resulted in being highly efficient for the recovery and infectivity preservation of viral bioaerosols. They were successfully used for direct evidence that SARS-CoV-2 can be viable in aerosols produced by infected people in hospitals rooms, representing a risk for transmission of the virus [16,52].

### 2.2. Air Sampling Strategy

Regarding the state of the art of the strategies for air sampling in specific contexts or for air sampling of other substances, the most relevant information obtained to guide the definition of the strategy for SARS-CoV-2 air sampling was extracted from standards issued by the International Organization for Standardization (ISO) and the European Committee for Standardization (CEN), primarily ISO 16000 (Part 1, Part 2, Part 5, Part 7, Part 12, and Part 15) [53,54,55,56,57,58] and EN 689 [59] on sampling strategies of specific indoor airborne pollutants and on measurement of exposure by inhalation to chemical agents. In addition, some of the reports issued by the Commission of the European Communities were found to be of interest: ECA Report n.6 [60] and ECA Report n.12 [61] on indoor chemical and biological pollution and its impact on people. Finally, other significant documents were reports issued by Istituto Superiore di Sanità (an agency of the Italian national health system) containing specific indications for the sampling of chemical and biological pollutants in the living and work environments [28,62,63].

The above literature has been analysed in order to identify and characterise the factors declining who, what, where, how, how many, when and how long to sample, and a set of specifications for each factor guiding the setup of a sampling plan, based on the characteristics and criticalities of the environment [64].

In addition to the above references, the scientific literature was analysed to confirm and, in some cases, to disambiguate and complete the specifications for the identified factors. For example, with reference to where to measure, one of the specifications needed is the height at which to position the sampler. For preschools, the context of the case study of this paper, the Istituto Superiore di Sanità indicates that the sampling height should be 1 m for indoor air quality monitoring [63]), and between 1 and 1.2 m for volatile organic compounds (VOCs) monitoring [65]. Similar heights are also adopted by several scientific studies dealing with sampling of bacteria [66], persistent organic pollutants (POPs) [67], and chemical parameters such as PM_10_, PM_2.5_, PM_1_, CO_2_, and CO [68] in preschools.

Further insights from the scientific literature have focused on the modes of SARS-CoV-2 transmission and infection that affect, for example, sampling point selection and sampling frequency. In particular, occupants’ proximity and ventilation are mentioned as key determinants of transmission risk [7,8]; face mask wearing and social distancing are identified among risk reduction measures [69,70]; the activities carried out affect the emission rate of virus-laden particles [71]; and the number of occupants and their time spent in a room are issues to be limited for mitigating airborne transmission [72]. Consequently, all of these issues affect the likelihood that the virus is present in the air and therefore detectable.

### 2.3. Case Study

In order to test the applicability of the sampling strategy in real environments, to verify its completeness and to evaluate its effectiveness in guiding the definition of a sampling plan, a case study has been carried out in kindergartens. The case study also enabled fine-tuning of certain aspects of the strategy, in particular with regard to the information to be collected during the preliminary investigation and the information to be recorded during sampling in order to support the interpretation of the results.

In Italy, during the period in which the case study was developed, kindergartens were an interesting context for the case study for several reasons.

From the re-opening of Italian schools in September 2020, the Government imposed different prevention measures depending on the age of the students, like social distance, frequent hands cleaning, and face masks. In kindergartens, children were exempt from wearing face masks, and it was more difficult to maintain social distancing, making them possible spreaders of current and emerging variants [73]. Instead, teachers constantly wore face masks and adopted other prevention measures. People with flu-like symptoms or a body temperature above 37.5 °C were not allowed to enter indoor premises.

Moreover, in kindergartens, as in schools, the vaccination rate was quite low and infections reached a high incidence, as documented by the high number of quarantined classes [74,75].

Two kindergartens with a total of four buildings in Milan, in the north of Italy, were considered in this case study:

1. the “Immacolata Concezione” institute (in the following named “Immacolata”), a private kindergarten located in via Elba 18, in a single building shared with a primary school, and
2. the “Luciano Manara” institute (in the following named “Manara”), a public kindergarten with three buildings located in Via Marx 2, Via Lamennais 19, and Via Airaghi 40 (in the following named “Marx”, “Lamennais” and “Airaghi”, respectively).

## 3. Results

### 3.1. The Sampling Strategy

The factors and specifications representing the sampling strategy for the presence of airborne SARS-CoV-2 are shown in Table 1.

In order to set-up the sampling plan for a specific application case on the basis of the proposed strategy, a preliminary investigation should first be carried out. The purpose of the preliminary investigation is to gather the following necessary information to decline the factors of the strategy:

- the layout of the building covered by the sampling plan with indication of dimensions of each room;
- the type of building and occupants (intended utilisation) of each room;
- the level and timing of occupancy and any occupiable positions of each room;
- the activities carried out in each room;
- the characteristics of the natural ventilation (presence, position, dimensions, frequency and opening time of doors and windows);
- the characteristics of the mechanical ventilation (presence and position of air conditioning system inlets and outlets, outdoor air flow rate, recirculated air flow rate, presence and position of fan-coils/room air conditioners, operating time);
- the rooms and the activities with no or discontinuous use of face masks or with use of inadequate face masks;
- and the CO_2_ concentrations, air temperature and air relative humidity, whether available.

During the sampling activities, it is necessary to record information supporting the interpretation of the sampling results. In particular, for each room where the sampling is carried out:

- the identification of the room;
- the name of the operator(s) performing the sampling activities;
- the identification of the measuring instrument used;
- and the settings of measurements/sampling volume (if the same for all measurements);

Other information should be recorded for each sampling/measurement point:

- a sample number/code;
- the location of the sampling point (room, location in room, height);
- the date, start time and end time of sampling;
- the settings of measurements/sampling volume (if specific to each measurement);
- the actual conditions of the room during sampling (e.g., occupancy levels, type of occupants, activities carried out, ventilation conditions, use of face masks);
- any additional parameters sampled (e.g., CO_2_ concentrations, and air temperature and relative humidity);
- and the outdoor meteorological conditions.

As the operator(s) performing the sampling activities could either be exposed to SARS-CoV-2 and contaminate the sampled environment, they must wear all necessary personal protective equipment (PPE) and be adequately trained.

Finally, it must be taken into account that if samples cannot be sent immediately to the analytical laboratory, they must be stored in a refrigerator (+4 °C) for no longer than 48 h or at −80 °C if the sample cannot be processed within 48 h [76]. Requirements for packaging, labelling and transport of biological samples may be defined by the legislation of different countries.

With reference to the sample analysis phase, in addition to the results in terms of positivity or negativity, information on the type of analysis performed and, where available, the SARS-CoV-2 variant detected should be recorded.

### 3.2. The Case Study

#### 3.2.1. Preliminary Investigation

With the preliminary investigation, the headteachers of the two kindergartens provided the building layouts, with dimensions and intended utilisation of the rooms, and area of the windows. In particular, the Immacolata building has three floors and a basement, while the three Manara buildings have only one floor. Where data were not detailed enough due to missing information regarding window dimensions and room heights, they were measured in the field for Manara buildings (Marx and Airaghi), while for Immacolata they were extracted from a Building Information Model (BIM) developed thanks to a former research project [77,78]. Thanks to the same project, Immaculata had a system for detecting CO_2_ concentration, air temperature and air relative humidity in all classrooms and some common areas; in particular, permanent sensors were in the upper part of the classrooms to provide teachers and kindergarteners with CO_2_ values and to suggest opening the windows. None of the buildings were equipped with mechanical ventilation.

The headteachers were also interviewed to acquire the level and timing of occupancy of the rooms.

#### 3.2.2. The Sampling Plans

Based on the developed strategy and on the information gathered through the preliminary investigation, a sampling plan was set-up for each of the four school sites involved in the case study. As an example, Table 2 shows the sampling plan for Immacolata.

**Figure 1:**
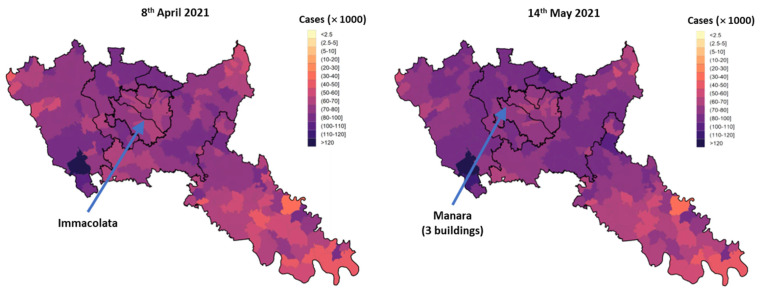
Maps of the infections monitored by the Unit of Epidemiology of the Health Protection Agency of Milan (which covers the City of Milan and the Province of Lodi).

The sampling campaign was held in spring 2021 (7 April 2021 at Immacolata and 13 May 2021 at Manara) when the city of Milan was registering a high number of confirmed COVID-19 infections. Figure 1 shows the locations of the schools on the map of infections on the periods when the sampling activities were carried out. Please note that the dates on the maps do not correspond exactly to the sampling dates because the maps were updated every two days.

**Figure 2:**
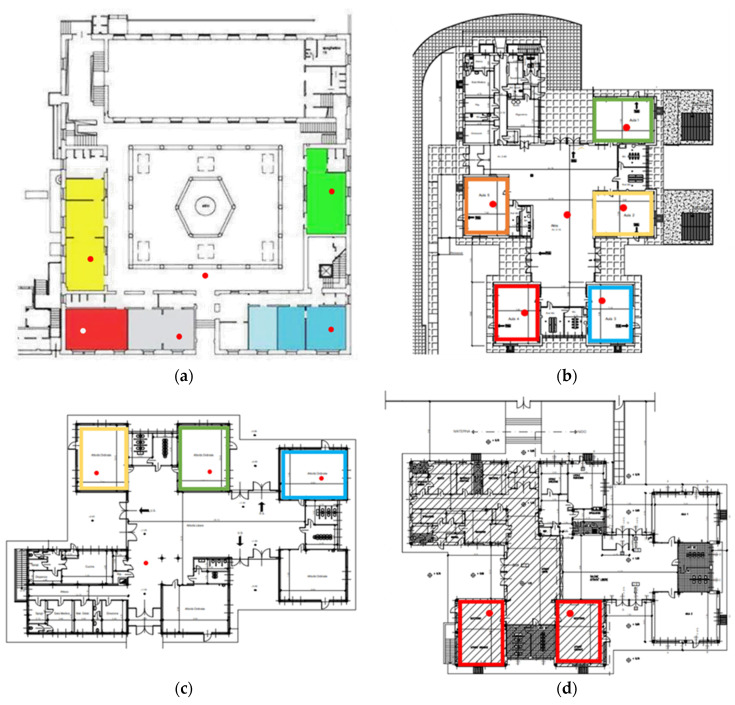
(**a**) Ground floor of Immacolata building; (**b**) Marx building; (**c**) Airaghi building; (**d**) Lamennais building.

At Immacolata, 9 indoor environments (7 classrooms with a total 89 children and 16 teachers, 1 atrium and 1 dining hall) were tested, while at Manara 12 indoor environments: 5 classrooms (with 68 kindergarteners and 11 teachers) and 1 atrium at Marx; 3 classrooms (with 45 kindergarteners and 5 teachers) and 1 atrium at Airaghi; and 2 classrooms (with 34 kindergarteners and 4 teachers) at Lamennais. Surface sampling was additionally performed in the Immacolata dining hall where the lunch tables were considered high-touch and droplet deposition surfaces. Figure 2 shows the rooms and the sampling points in the four buildings.

Operators took note of all the characteristics of ventilation and occupancy during the whole sampling. Air temperature, relative humidity, and CO_2_ concentrations were also collected. At Immacolata, the measures of these parameters in the classrooms and dining hall were performed with a set of Aranet4 Pro sensors coupled with an Aranet Pro base station, developed by SAF Tehnika J.S.C. (Riga, Latvia), integrated into a centralised data collection system developed by the eLUX Laboratory of the University of Brescia in the context of a former research project [77,78]. Moreover, in each classroom, a light indicator warns teachers for CO_2_ concentrations higher than 700 ppm and suggests opening the windows. For one of the classrooms, the data were not gathered due to a temporary unavailability of the sensors. The atrium was not monitored being only a passageway. At Manara, instead, the corresponding Aranet4 portable device was used for all the rooms.

Air sampling was carried out with a Coriolis μ air sampler, developed and provided by Bertin Technologies S.A.S. (Montigny-le-Bretonneux, France), which is conceived for bio-contamination control and based on the cyclonic technology. The Coriolis μ has an air volume flow rate ranging from 100 to 300 L/min. With reference to this parameter, it is known that high air flow rates may affect the viability of viruses [8,44,50]. In our case, this is not relevant since the presence of SARS CoV-2 is checked with RT-PCR and not with microbiological culture. For this reason, we prioritised high sampling volumes setting an air flow rate of 300 L/min for 20 min. Moreover, the Coriolis μ can collect particles in size range higher than 0.5 μm in a cone filled with a liquid up to 15 mL. In our case, the cones were filled with 15 mL of RNase free water at the beginning of the sampling, reaching a minimum volume of about 5 mL at the end. Polyester swabs (13 mm head width, 4.2 mm head thickness and 25.7 mm head length), developed and provided by BIOSIDE S.r.l. (Lodi, Italy), were used for surface sampling. After their fractioning, the swabs were stored in a neutral transport medium (PBS pH 7.4).

In order to ensure the representativeness of the sampling, children and their teachers carried out their usual activities and were allowed to approach the sampler without touching it. The operators performing the sampling activities waited outside the room after positioning and setting up the instrument.

All samples were stored at 4 °C using dry ice for a maximum of 24 h prior to the analyses.

#### 3.2.3. The Sample Analyses

Real Time Polymerase Chain Reaction (RT-PCR) analysis was performed on all the liquid samples of the Coriolis µ, and surface swab samples. All the samples were analysed in two different laboratories (BIOSIDE and AAT) for confirmation, using the qualyfast^®^SARS-CoV-2 Multiplex 2 One Step real time PCR kit (BIOSIDE) according to manufacturer’s instructions. Final reactions of 15 µL were formed by mixing 5 µL extracted RNA and 10 µL RNase free water to rehydrate the lyophilised detection kit. The kit provides amplification of two specific RNA sequences belonging to the SARS-CoV-2 (S gene and RdRP gene), a specific sequence of sarbecovirus (Charitè Protocol, E gene), and simultaneous co-amplification of RNA-IAC (RNA Inhibition Amplification Control). This approach is used to highlight a possible effect of inhibition in the RNA from the sample.

At BIOSIDE, extraction was performed from 400 µL of environmental samples or PBS using Sera-Xtracta Virus/Pathogen Kit (Cytiva, Marlborough, MA, USA) with King Fisher 96 (Thermo Fisher Diagnostics, Waltham, MA, USA) according to the instruction for use. RNA was eluted with 60 μL of water in the final step. RT-qPCR was performed on a CFX96 real time PCR machine (Bio-Rad, Hercules, CA, USA) with the following cycle: 1.50 °C for 30 min; 2.95 °C for 10 min; 3.95 °C for 15 s; 4.58 °C for 30 s. Results interpretation and Ct calculation were performed with Bio-Rad CFX Manager IDE software (Bio-Rad). Targets detected with a Cq less than 40 were considered positive. A sample was considered positive if at least one of the targets sought is positive.

At AAT, extraction was performed from 400 µL of samples using CommaXP Virus DNA/RNA extraction kit (Biocomma, Shenzhen, China), according to the manufacturers’ instructions. RNA was eluted with 60 μL of water in the final step. RT-qPCR was performed on a StepOnePlus (Applied Biosystems, Thermo Fisher Diagnostics, Waltham, MA, USA) with the same cycle and procedure for identification of positive samples used at BIOSIDE.

#### 3.2.4. The Sampling Results

The sampling results, together with the occupancies, the volumes, and the measured additional parameters, are reported in Table 3.

All the air samples collected by Coriolis μ and analysed by RT-PCR were negative with respect to the presence of SARS-CoV-2. Surface samples collected through swabs were also negative.

Data concerning the possible SARS-CoV-2 positivity of children and teachers present during the sampling campaign were not available.

Sampling was performed in two sunny spring days in April and May when the weather in northern Italy is quite variable. The average outdoor temperature in Milan was approximately 7.8 °C on 7 April and 17.4 °C on 13 May, as measured by the Regional Agency for Environmental Protection (ARPA) [78].

**Figure 3:**
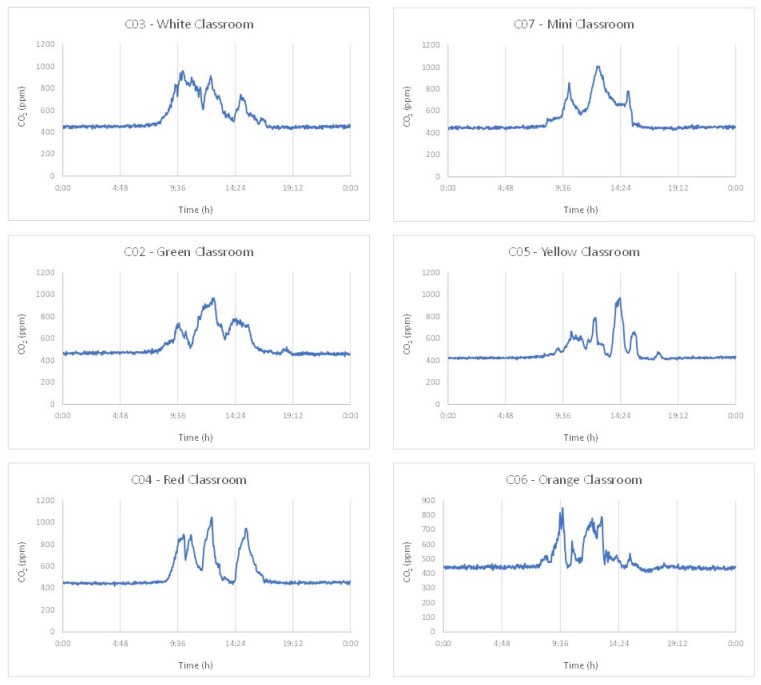
CO_2_ trends in the classrooms at Immacolata (permanent sensors).

With reference to CO_2_, the concentrations vary significantly over time based on the occupancy and ventilation conditions of the classrooms. This can be highlighted looking at CO_2_ trends at Immacolata in Figure 3. Compared to the background value (440–450 ppm), increasing trends are observed when the classrooms are occupied and with low or no ventilation, while decreasing trends correspond to periods when windows are open and/or when the children leave the classrooms.

In most of the classrooms at Marx and Airaghi, all the windows were partially kept open whole time, with CO_2_ levels well below the peaks measured at Immacolata.

Finally, during the samplings at Lamennais, children and teachers were having lunch in their classrooms. The concentrations are quite different because windows were closed in I08 and partially opened in I09.

## 4. Discussion

**Figure 4:**
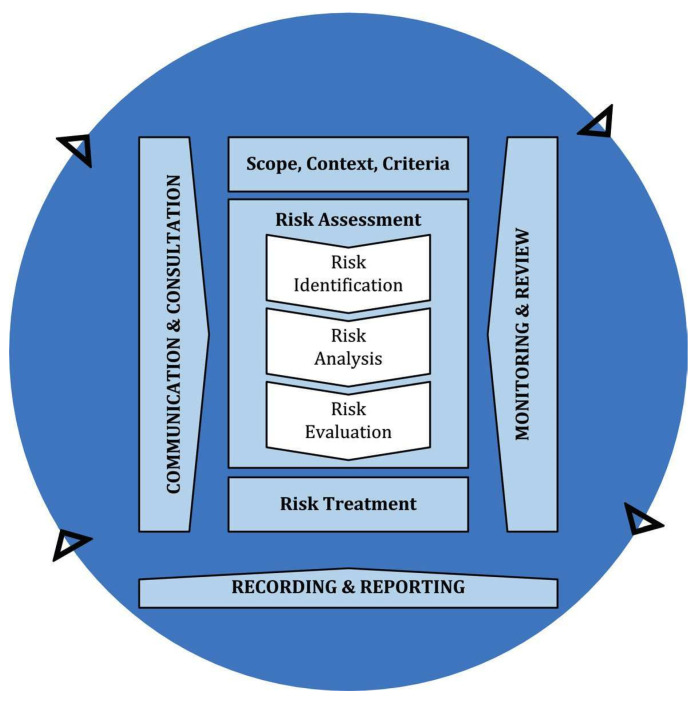
Risk management process. Reprinted with permission from [79]. 2018, UNI Ente Italiano di Normazione.

The developed strategy for airborne SARS-CoV-2 detection can be contextualised within the broader management process of the risk of virus transmission. As shown in Figure 4, a risk management process “involves the systematic application of policies, procedures and practices to the activities of communicating and consulting, establishing the context and assessing, treating, monitoring, reviewing, recording and reporting risk” [17]. This process is iterative and supports organisations in making informed decisions.

In this context, our strategy aims to be a solid pillar on which to base the risk identification phase of airborne SARS-CoV-2 infection.

With regard to the case study, as reported in the sample results section, the tested air samples were negative. In general, non-detection of SARS-CoV-2 in samples could be attributed to several causes: (a) the absence of infected individuals within the environment, (b) the effectiveness of the use of face masks in containing droplet and aerosol emissions, (c) the effectiveness of natural and/or mechanical ventilation in “cleaning” the air, and (d) the ineffectiveness of the sampling and analysis chain in detecting the presence of the virus in the air, due either to its poor design or to its faulty implementation.

With reference to point (a), as stated above in our case, no information was available on the possible presence of infected persons. In fact, the lack of such information is precisely the scenario for which the strategy was devised. However, the occupants of the rooms were all asymptomatic and the tests carried out on the surface swabs were also negative. It should also be noted that surface sampling is the elective mode that public health agencies, primarily the WHO [22], indicate to use for environmental sampling of SARS-CoV-2, and for which they also provide relevant sampling strategies. The asymptomatic nature of occupants and the negativity of surface swabs does not allow the presence of positive individuals to be excluded but reduces their likelihood.

The use of face masks—point (b)—is considered one of the most effective prevention measures because of their ability to reduce the spread of aerosols and droplets containing the virus. In our case study, only teachers wore them. In Italy, kindergartners were exempted from wearing them. Therefore, this preventive measure had only a minor influence on the non-detection of the virus in the air.

As already mentioned, the ventilation of indoor environments—point (c)—is considered one of the key factors in determining the risk of infection, since it affects the possible presence of the virus in the air and therefore also the possibility of detecting it. In the rooms of the case study, ventilation was only natural, not continuous, and highly variable both among the different rooms and between the dates when the sampling activities took place. Therefore, while it is possible that ventilation reduced the probability of detecting the virus, this effect would be limited since ventilation was discontinuous.

The last possible cause (d) is related to the poor design of the air sampling and sample analysis chain or its faulty implementation. In our case, the chain was previously validated in the laboratory using synthetic single strand RNA material. Moreover, the samplings and the analyses were carried out by the same experienced researchers who validated the chain in the laboratory. Consequently, this cause is considered irrelevant. However, it should be noted that any air sampling and sample analysis chains that differ in terms of type of sampler, measurement parameters, type of sample analysis, etc. will also require prior validation. At the same time, the operator(s) performing the sampling and analysis activities will have to be adequately trained for the purpose.

Having said that, it should be noted that not detecting the presence of the virus does not affect the achievement of the objective of our work. In fact, the objective was not to detect the airborne virus, but to define a strategy to guide the planning and carrying out of sampling activities in a relatively unexplored field. From this point of view, the proposed strategy proved to be fit for purpose. The conducted case study only involved the need for a fine-tuning of certain aspects of the strategy, which was consequently defined in its final version. At the same time, the case study allowed us to test the applicability of the strategy in a real environment, verifying its completeness and assuring its effectiveness in guiding the definition of sampling plans.

Finally, the strategy will not only allow other studies to be addressed for the currently identified SARS-CoV-2 variants, but also to be repeated early and rapidly as new variants and even new airborne viruses emerge and spread. This will help to increase the resilience of working and living environments. Consequently, the value of the proposed strategy extends beyond the current pandemic period.

## 5. Study Limitations and Future Research

The developed strategy was tested in a case study involving several environments of the same type and located close to each other. However, a full validation of the strategy will require conducting case studies in real environments of different types and in different geographic locations, as well as under controlled conditions, i.e., in environments with the actual presence of SARS-CoV-2 or synthetic single strand RNA material in the air. For such validation, it is furthermore important that the strategy is also tested by other researchers and practitioners.

Another limit of this study concerns the fact that the field test revealed some factors in the strategy that will benefit from more precise specifications: the number of sampling points and their location in space. With regard to these two factors, the proposed strategy provides specifications that need to be interpreted. In order to avoid excessive arbitrariness in defining sampling plans, further studies should be developed to quantitatively investigate aspects that influence the spread of the virus in the air (e.g., level of occupancy, time of stay of occupants, flows of people, intensity of activities carried out, ventilation conditions, and use of face masks). In our case, this potential limit of the strategy did not have a relevant impact. Children and teachers in kindergarten rooms do not occupy fixed positions for long periods of time, but move widely and frequently, thus contributing to uniform air conditions. Consequently, even if the sampling plan had provided for a different number or location of sampling points, the sampling results would most likely have been the same.

Beyond the future developments highlighted so far, further studies must be carried out to support the entire risk management process, including the phases of risk analysis, risk evaluation, and risk treatment. Indeed, these three phases involve detailed considerations of uncertainties, consequences, likelihood, scenarios, risk criteria, risk treatment options, and their effectiveness, based on available and reliable information.

The possible future adoption and dissemination of our strategy by standardisation bodies and/or health authorities may lead to the widespread use of air sampling as a risk identification tool, as well as to the uniformity of the sampling plans implemented and of the results obtained. The gathering of sampling results obtained according to the same standard may provide a broad and shared basis of information for future studies aimed at developing considerations and tools to support the remaining steps of the risk management process.

With regard to the risk analysis phase, future studies could focus on correlations between the results of sampling and factors such as the viral load of the infected persons, their use of face masks and the activity they carry out, the level of occupancy of the rooms, and the time of stay in the rooms and the ventilation conditions. Secondly, the adoption of sample analysis techniques capable of not only detecting the presence of the virus in the air but also of quantifying its concentration may enrich the knowledge of these correlations. In addition, it may lead to the possible definition of concentration action levels to guide the risk evaluation and risk treatment phases.

Once a full validation of the strategy has been carried out and the next phases of the risk management process have been studied, it may be possible to assess whether to adopt indoor air sampling as a public policy approach to control the spread of the virus. The necessary resources and consequently the applicability in low- and middle-income countries are significant aspects that should also be considered.

Another research direction could be to further study the use of CO_2_ concentration as a proxy for risk of infection. In particular, this will be possible once a wide set of sampling results obtained according to the same standard will provide enough data to carry out a multivariate analysis.

## 6. Conclusions

In this work, we propose a strategy to set-up indoor air sampling plans for the detection of SARS-CoV-2. The strategy identifies and characterises the factors declining who, what, where, how, how many, when and how long to sample, and a set of specifications for each factor guiding the setup of a sampling plan, based on the characteristics and criticalities of the indoor environment to be investigated.

A case study conducted in two kindergartens proved its completeness, effectiveness, and suitability as a key tool in the airborne SARS-CoV-2 infection risk management process, in particular for the phase of risk identification.

Since the strategy can be a standard, it can not only guide practitioners in designing and performing air sampling activities, but also allow for consistent results from samplings carried out in different environments, conditions, and geographic locations. The results thus obtained may provide a broad and shared basis of information for future studies aimed at developing considerations and tools to support the phases of risk analysis, evaluation, and treatment.

## Figures and Tables

**Table 1:** Sampling strategy for airborne SARS-CoV-2.

Factor	Specifications	
Time of the year	Primarily in the periods with the highest incidence of infection and/or with reduced or eliminated protective measures based on personal behaviours and/or testing	
Additional parameters to be sampled, whether available	CO_2_ concentrations, and air temperature and relative humidity [10]	
Conditions during sampling: Activities, occupancy, and use of face masks	Actual conditions of the indoor environment	
Conditions during sampling: Ventilation	Actual conditions of the indoor environment	
Sampling frequency	At least one sample at each sampling point; two samples, if possible If presence of the virus over time is to be verified, define in relation to the following: level of occupancy [7,8,69,70,72] time of stay of occupants [72] flows of people [7,8,69,70] intensity of activities carried out [71] ventilation conditions [3,7,8,9] use of face masks [3,9,69,70]	
Sampling duration	Depending on sampler specification (e.g., for Coriolis μ sampler: at least 20 min) [27,30,31,32,33,35,36,41]	
Sampler: Type and air volume flow rate	Coriolis μ:	Prefer high flow rates in large and highly ventilated environmentsPrefer low flow rates to preserve viability
	Other samplers:	Depending on sampler specifications [27,30,31,32,33,35,36,41]
Sampling points: Rooms in the building	Primarily the rooms where the following conditions occur: high level of occupancy [7,8,69,70,72] long stay of occupants [72] high flows of people [7,8,69,70] high intensity of activities carried out [71] poor ventilation [3,7,8,9] lack or discontinuous use of face masks or use of inadequate face masks [3,9,69,70]	
Sampling points: Location in the room	Primarily, the points in the room near where the following conditions occur: high level of occupancy [7,8,69,70,72] long stay of occupants [72] high flows of people [7,8,69,70] high intensity of activities carried out [71] poor ventilation [3,7,8,9] lack or discontinuous use of face masks or use of inadequate face masks [3,9,69,70] At least 1 m away from walls, and at least 0.5 m away from heat sources and from openings (doors and windows) or mechanical ventilation inlets (fan-coils and air conditioning system inlets) [53,54,55,56,57,58,59,63,65,66,67,68]	
Sampling points: Height	At the height of the average breathing zone, depending on the predominant posture of the occupants (children, adolescents, adults). General indications [53,54,55,56,57,58,59,63,65,66,67,68]: preschools: about 1 m above the floor schools: 1–1.5 m above the floor in classrooms, or 1.5 m above the floor in common areas offices (and similar): about 1.2 m above the floor for sitting, or 1.5 m above the floor for standing means of transport: more than 1.2 m above the floor	
Parallel investigations	Complementary surface sampling [22]	

**Table 2:** Sampling plan for Immacolata.

Factor	Specifications
Time of the year	Spring (due to the high number of infections)
Additional parameters to be sampled, whether available	CO_2_ concentrations, air temperature, and air relative humidity (only in classrooms and dining hall)
Conditions during sampling: Activities, occupancy, and use of face masks	Arrival of kindergarteners with parents in the atrium. Typical kindergarten activities in the classrooms and lunch in the dining hall. The kindergarteners have lunch in two shifts. FFP2/N95 face masks worn only by teachers
Conditions during sampling: Ventilation	Window opening when kindergarteners leave the room for outdoor activities and when the CO_2_ monitoring system warns teachers for high concentrations. No mechanical ventilation available
Sampling frequency	One sample at each sampling point
Sampling duration	Classrooms: 20 min. Atrium: 60 min
Sampler: Type and air volume flow rate	Coriolis μ: 300 L/min
Sampling points: Rooms in the building	Atrium, Green classroom, White classroom, Red classroom, Yellow classroom, Orange classroom, Mini classroom, Spring classroom, dining hall
Sampling points: Location in the room	Atrium: one point along the entrance/exit path. Classrooms: one point among the desks. Dining hall: one point among the dining tables
Sampling points: Height	About 1 m (with the sampler located on a trolley)
Parallel investigations	Swab surface sampling (only in the dining hall)

**Table 3:** Sampling points: room volumes, occupancies, sample results, and additional parameters measured in the rooms. The values of air temperature (T), air relative humidity (RH), and CO_2_ concentration are averaged over the sampling duration.

Building	Point	Volume (m^3^)	Occupation	Sample Result	T (°C)	RH (%)	CO_2_ (ppm)
Immacolata	C01—Atrium	2452	Variable	Negative	n.a.	n.a.	n.a.
	C02—Green Classroom	213	2 adults + 13 kids	Negative	19.4	31.0	577
	C03—White Classroom	197	3 adults + 14 kids	Negative	21.9	23.8	899
	C04—Red Classroom	183	2 adults + 17 kids	Negative	19.3	25.9	840
	C05—Yellow Classroom	252	2 adults + 15 kids	Negative	19.4	19.0	598
	C06—Orange Classroom	240	3 adults + 15 kids	Negative	22.7	13.0	598
	C07—Mini Classroom	241	2 adults + 9 kids	Negative	20.6	25.6	780
	C08—Spring Classroom	178	2 adults + 6 kids	Negative	n.a.	n.a.	n.a.
	C09—Dining room	353	2 adults + 29 kids	Negative	18.7	25.8	747
	C10—Dining room	353	2 adults + 30 kids	Negative	17.8	15.2	548
Marx	I01—Atrium	n.a.	Variable	Negative	15.1	64.3	444
	I02—Green Classroom	210	2 adults + 16 kids	Negative	18.8	55.8	642
	I03—Yellow Classroom	207	2 adults + 14 kids	Negative	19.4	54.5	518
	I04—Blue Classroom	210	2 adults + 17 kids	Negative	20.5	53.8	604
	I05—Orange Classroom	202	2 adults + 9 kids	Negative	20.8	53.0	586
	I06—Red Classroom	206	3 adults + 12 kids	Negative	20.4	52.4	536
Lamennais	I08—N.1 Classroom	208	2 adults + 17 kids	Negative	24.6	45.8	888
	I09—N.2 Classroom	207	2 adults + 17 kids	Negative	22.2	48.8	601
Airaghi	I11—Blue Classroom	209	2 adults + 12 kids	Negative	22.0	44.4	453
	I12—Yellow Classroom	209	2 adults + 17 kids	Negative	21.7	46.2	632
	I13—Green Classroom	209	1 adults + 16 kids	Negative	22.3	45.2	565
	I15—Atrium	n.a.	Variable	Negative	22.5	42.2	448

## Data Availability

Not applicable.
